# Investigating Therapeutic Potential of *Trigonella foenum-graecum* L. as Our Defense Mechanism against Several Human Diseases

**DOI:** 10.1155/2016/1250387

**Published:** 2016-01-18

**Authors:** Shivangi Goyal, Nidhi Gupta, Sreemoyee Chatterjee

**Affiliations:** Department of Biotechnology, The IIS University, Gurukul Marg, SFS, Mansarovar, Jaipur, Rajasthan 302020, India

## Abstract

Current lifestyle, stress, and pollution have dramatically enhanced the progression of several diseases in human. Globally, scientists are looking for therapeutic agents that can either cure or delay the onset of diseases. Medicinal plants from time immemorial have been used frequently in therapeutics. Of many such plants, fenugreek is one of the oldest herbs which have been identified as an important medicinal plant by the researchers around the world. It is potentially beneficial in a number of diseases such as diabetes, hypercholesterolemia, and inflammation and probably in several kinds of cancers. It has industrial applications such as synthesis of steroidal hormones. Its medicinal properties and their role in clinical domain can be attributed to its chemical constituents. The 3 major chemical constituents which have been identified as responsible for principle health effects are galactomannan, 4-OH isoleucine, and steroidal saponin. Numerous experiments have been carried out* in vivo* and* in vitro* for beneficial effects of both the crude chemical and of its active constituent. Due to its role in health care, the functional food industry has referred to it as a potential nutraceutical. This paper is about various medicinal benefits of fenugreek and its potential application as therapeutic agent against several diseases.

## 1. Introduction

Many chemicals are encountered by human either accidentally because they are in the atmosphere or by contact during occupational and recreational activities or by ingestion of food additives. It is conceivable that some chemicals may be inadvertently released into the environment and therefore be injurious to human health. With the increasing rate of use of industrialization and diesel exhaust emission and cigarette smoke; it is obvious that the chemicals are capable of producing undesirable effects on biological tissues. The air pollution index correlated with respiratory and digestive tract and urogenital, blood and skin cancer. Air pollution was estimated to account for 80% of premature deaths, while 14% of death rate was due to chronic diseases according to data provided by World Health Organization (WHO). Evaluation by WHO's International Agency for Research on Cancer (IARC) in 2013 found out the close connection between outdoor air pollution, specifically particulate matter, and increased cancer incidence.

A number of environmental chemicals, both man-made and of natural occurrence, are under strong suspicion as carcinogens, which are important in the etiology of the cancer. Polycyclic aromatic hydrocarbons (PAHs) are ubiquitous environmental pollutants and represent one of the few clearly defined classes of chemicals responsible for the development of skin cancers. Humans are constantly exposed to PAHs through polluted air, cigarette smoke, automobile exhaust, and other airborne pollutants [1]. Polycyclic hydrocarbons vary in their carcinogenic potencies; for example, the compound dibenz[a,c]anthracene has very little carcinogenic activity [2]. Among the most potent hydrocarbon carcinogens are 3-methylcholanthrene and 7,12-dimethylbenz[a]anthracene (DMBA) [3]. DMBA serves as a tumor initiator by making necessary mutations in chemically induced skin tumors in mice. This DMBA, a polycyclic aromatic hydrocarbon, is currently the most frequently utilized initiating agent but additional agents can serve as chemical initiators. Systemic exposure of DMBA is generally effective; however, it is often applied topically to induce skin carcinogenesis in two-stage skin papillomagenesis murine models [4].

Among several different types of cancers known, skin cancer is found to be the most uncommon malignancy in the world; however, the rate of increase in skin cancer over the years has been observed to be progressive [5]. According to the survey, there has been a 65% rise in the incidence since 1980. In the United States, more than 33% of the cancers are skin cancers, while statistics in India account for 1-2% of diagnosed cancers. Researchers have reported basal cell carcinoma as the common form of skin cancer worldwide, while squamous cell carcinoma is known to be the most prevalent form of skin cancer in India. Skin cancer can be divided into two forms: nonmelanoma skin cancers (NMSCs) and melanoma skin cancers (MSCs). Both squamous cell carcinoma (SCC) and basal cell carcinoma (BCC) come under the heading of NMSCs [6].

Various studies have been done by researchers around the world to find out strategies for preventing and treating cancers. Traditional therapies such as radiation, chemotherapy, and surgery have been in use but severe side effects often force patients not to opt for these. For incidence, treatment against breast cancer utilized systemic chemotherapy and failed to give satisfying results [7]. Similarly, usage of doxorubicin as a chemotherapeutic agent against breast cancer treatment not only was less effective but also was toxic to normal tissues [8]. Thus chemoprevention provides a promising strategy to combat dreadful diseases like cancer, several adverse effects such as osteoporosis, and degenerative diseases [9]. Prevention, inhibition of progression and reversal of the normal physiological conditions by the use of natural products, biological products, or synthetic agents, is called chemoprevention. These natural products/extracts in form of day-today dietary components possess the potential of reducing the toxic effects rendered by chemotherapy. According to the epidemiological study, consumption of fruits and vegetables resulted in 50% decrease in cancer development and incidence. F'guyer in his study suggested phytochemicals such as curcumin, green tea extracts, gingerol, and quercetin which could fight against the reactive oxygen species (ROS) of skin and can help in treatment against skin carcinogenesis [10]. With onset of wider research involving preclinical and clinical observations in chemoprevention, valuable data have been generated. This could be beneficial in preventing the onset of disease and suppressing its progress and developing an outlook towards chemoprevention as a challenging and rational strategy of future. Earlier discoveries involved isolation of anticancer drugs from plants such as* Catharanthus roseus, Camptotheca acuminate, and Taxus brevifolia*. Some of these are presently used as potential anticancer drugs like Taxol, Vincristine, Vinblastine, and Camptothecin [11]. Citrus peel extract containing phenolic and flavonoid compounds has been known to be potential natural antioxidant [12]. Multipurpose approaches of the plants offering antioxidant, anti-inflammatory, antidiabetic, and other roles have thus rendered them as the potential and indispensable candidate for the drug discovery without harming the normal physiology of the human body.

One of the traditionally known plants is* Trigonella foenum-graecum* (L.) (fenugreek). It grows once a year and is a self-pollinating plant. Species of* Trigonella* are widely distributed throughout the world. The plant has been mainly found on the continents of Asia (India and China), parts of Europe, Africa, Australia, and North and South America [13]. It bears two cotyledons and belongs to subfamily Papilionaceae, family Leguminosae (Fabaceae). The genus,* Trigonella*, is a Greek word which means “three angled” and the word fenugreek which is derived from* foenum-graecum* means Greek hay. Presence of complex array of important phytochemicals renders fenugreek as one of the important medicinal plants. The leaves and seeds of methi (Indian name) have been used extensively to prepare extracts and powders for medicinal uses [14]. Scientists have reported several medicinal uses of fenugreek seeds such as remedies for diabetes and hypercholesterolemia, hepatoprotective protection against free radicals, and protection against breast and colon cancer [15]. These protective roles are possible due to the nonnutritive secondary metabolites also known as phytochemicals. The major constituents that are present in fenugreek seeds are carbohydrates, proteins, lipids, alkaloids, flavonoids, fibers, saponins, steroidal saponins, vitamins, and minerals, nitrogen compounds which can be categorized under nonvolatile and volatile constituents [16].

## 2. Fenugreek as Potential Therapeutic Agent against Several Diseases

Apart from the usage in bakery products, frozen dairy products, condiments, spices, pickles, and beverages, fenugreek is known to have numerous beneficial health effects. Gastric ulcers can easily be treated by fenugreek seeds. The seed oil acts as an emollient and makes skin smoother and soft. The cleansing action of fenugreek makes it a valuable plant as it helps purify blood, cleaning lymphatic system, and detoxify the body. In diseases like hay fever and sinusitis it can be used. The seeds are considered useful in heart disease and aphrodisiac and as a galactogogue promoting lactation [17]. Different regions in the world use fenugreek for different purposes; for example, in China, seeds are used to treat cervical cancer and for kidney problems. The aerial parts of plant are used to treat abdominal cramps during diarrhea in the Middle East and the Balkans. In southern India, roasted seeds are used as a treatment for dysentery. The smallpox patients are also given an infusion of seeds as a cooling agent. Being a natural health product, it is capable of treating and curing diseases, thus providing medical and health benefits. As a result of which, it has been considered a potential nutraceutical [18]. Apart from the traditional medicinal uses, fenugreek is found to have many pharmacological properties such as antidiabetic, antinociceptive, anticarcinogenic, antioxidant, anti-inflammatory, and hypocholesterolemic which are discussed below in detail. Tables 1(a) and 1(b) show different activities* in vivo* and* in vitro*, respectively.

### 2.1. Antidiabetic Activity

One of the chronic metabolic diseases is diabetes mellitus which occurs as a result of disordered metabolism of carbohydrates, proteins, and lipids. Though several forms of treatments are available in terms of medications and injectable insulin, they are accompanied with side effects. Diabetes mellitus can be regulated by the food habits which not only offer an economical approach but also are rich in chemical constituents that will help in maintaining blood glucose level. One of the well-studied herbal plants is fenugreek which has been quite researched with respect to its effect on diabetes. In one of the published studies by Raju et al., it is documented that seeds, leaves, and its extracts are a good agent in our fight against diabetes [19]. Xue et al., induced diabetes by streptozotocin in rats and effect of fenugreek water seed extract was determined via three different dose levels by intragastric intubation. It was observed that there was a weight gain in fenugreek treated mice as compared to the group that received only streptozotocin. In addition, blood glucose level decreased to a greater extent as compared to the group that received streptozotocin only [20]. Similar results were obtained in the study done by another group of researchers who found that there was an increase in the body weight of rabbits that were supplemented with fenugreek as compared to the alloxan monohydrate induced diabetic rabbits. Plasma glucose level was reduced by the oral administration of fenugreek seed powder not only in the diabetic rabbits but also in nondiabetic rabbits [21]. Ramesh et al. studied the effect of fenugreek seeds on alloxan induced diabetic rats. Histopathological analysis of pancreas of placebo controls was done in which normal acini and cytosol in the islets of Langerhans was observed. But there was an extensive damage to islets of Langerhans and reduced dimensions of islets in alloxan induced diabetes. Islets of Langerhans in diabetic rats that were treated with fenugreek extract were found to be restored [22].

An active compound can also be isolated from the crude extract which can perform a beneficial role against the glucose level. One such study was done by Moorthy et al. who isolated GII from the aqueous extract of fenugreek seeds. This isolated compound was able to reduce blood glucose in glucose tolerance test in subdiabetic and moderately diabetic rabbits. This isolated compound even showed better results than the standard tolbutamide [23].

Even in Egyptian folk medicine, fenugreek held an important place as a hypoglycemic agent. In an* in vitro* study being done by Gad et al., the extract of fenugreek in a dose-dependent manner was able to inhibit *α*-amylase activity. A further* in vivo* study concluded and confirmed* in vitro* inhibition as it showed suppression of starch digestion and absorption in normal rats, suggesting that the hypoglycemic effect of the used plant extract was mediated through insulin-mimetic effect [24].

Sharma observed the effects on the human patients who had reduced blood glucose concentration after the consumption of either the seeds or the leaves. Greater amount of reduction was observed using the whole seed followed by the gum isolated from cooked or uncooked seeds [25]. In another study by the same group, it was observed that, on consecutive consumption of fenugreek seeds, serum total cholesterol, LDL and VLDL cholesterol, and triglycerides were significantly reduced but no effect on HDL cholesterol levels was found [26]. The important constituents that are found to be responsible for generating the antidiabetic effects are galactomannan rich soluble fiber fraction, saponin, and an amino acid called 4-hydroxyleucine which helped in increasing insulin in hyperglycemic rats and humans [27].

### 2.2. Antioxidant Activity

Free radicals are being studied by the researchers for a long time as radicals are a source of ROS that hamper the structure of lipid membrane and thus initiate cascade of events leading to various diseases. To suppress generation of free radicals, natural products have been found as safe and effective remedy. One of the herbal extracts which is known to have antioxidant potential is fenugreek. Various studies have been done by the researchers to determine the antioxidant potential of fenugreek. Kaviarasan et al. conducted experiments on rat liver to evaluate the antioxidant potential of fenugreek seeds and it was found that methanolic seed extract was able to quench the free radicals [28]. This group in another set of experiments investigated the protective effect of fenugreek seed polyphenol extract. Rat liver was damaged using ethanol but when treated with fenugreek seed polyphenol extract (200 mg kg^−1^ day^−1^) there was a significant reduction in the levels of lipid peroxidation products and protein carbonyl content. Also there was an increase in the activities of antioxidant enzymes along with restoration of the levels of thiol groups [29]. Similar study was conducted by Thirunavukkarasu et al. in rats by using aqueous extract of fenugreek seeds. Ethanol was fed for 60 days to induce toxicity in rats which resulted in enhancement in the activities of serum aspartate transaminase, alanine transaminase, and alkaline phosphatase. However, simultaneously administrating aqueous extract of fenugreek seeds resulted in an increase of antioxidant level and prevented further rise in lipid peroxidation. The administration of aqueous seed extract could result in prevention of the enzymatic leakage and the rise in lipid peroxidation and enhancement of the antioxidant potential. Histopathological studies related to the rat liver and brain revealed the protective role of the seed extract against ethanol induced toxicity [30].

The constituents that are understood to be responsible were flavonoids and phenolic compounds which generally marks their presence in the polar solvent system due to their self-polar nature. Thus, due to the ability of fenugreek extracts to quench the radicals, it can be a useful candidate to alleviate the harmful effects of various diseases and thus can be used for treatment purposes.

### 2.3. Antitumor and Anticarcinogenic Activity

The chemical constituents of fenugreek possessing anticancer activity are phytoestrogens and saponins [31]. Saponins selectively inhibit cell division in tumor cells and also can activate apoptotic programs which can lead to programmed cell death [32]. In an* in vivo* study that was carried out on rats, azoxymethane was used to induce colon cancer. The effect of fenugreek seed powder along with its bioactive compound diosgenin was checked and it was observed that both the crude extract and diosgenin were able to inhibit the formation of aberrant crypt foci (ACF) which can be observed as preneoplastic lesion. After the positive response of the extract in* in vivo* experiment, anticancer potential of diosgenin was explored in* in vitro* experiments. HT-29 human colon cancer cells were used and it was seen that diosgenin inhibited the proliferation of cells along with the induction of apoptosis. The effect on apoptosis can be validated by observing the effect on apoptotic proteins. Diosgenin suppressed the expression of proapoptotic protein bcl-2 and there was an increase in the expression of caspase-3, an antiapoptotic protein [31].

Shishodia and Aggarwal reported diosgenin to have anticancer activity in bone cancer. It suppressed cell proliferation and development of bone cells through inhibition of tumor necrosis factor [33]. Protodioscin, a furostanol saponin isolated from fenugreek, also induces apoptotic changes leading to death in a leukemic cell line (HL-60) [34]. Several studies on anticancer properties of chemical constituents of fenugreek have been done and have shown positive results. Some constituent of alkaloids, called “trigonelline,” has revealed potential for use in cancer therapy [35].

*In vivo* cytostatic and cytotoxic effect of fenugreek seed extract was studied. Breast cancer in the mammalian model, that is, female Wistar rats, was induced by DMBA, a polycyclic aromatic hydrocarbon. Inhibition of the mammary hyperplasia and decrease in its incidence were seen after aqueous seed extract of fenugreek was given daily to the rats at a dose of 200 mg/kg b.wt for 120 days. Diosgenin was shown to suppress osteoclastogenesis which was induced by a cytokine, RANKL, through activation of NF-*κβ*. An increasing dose of diosgenin inhibited TNF-*α* activated transcription factors such as NF-*κβ* and Akt. Thus decrease in the cell proliferation by diosgenin was due to its inhibition on NF-*κβ* regulated gene products. The cell line used in the experiment was human chronic myelogenous leukemia (KBM-5) cells [36]. The ethanolic seed extract showed the antineoplastic effect against Ehrlich Ascites Carcinoma cells in mice. Intraperitoneal administration of the extract resulted in change in number and growth pattern of ascites cells and tumor growth was also seemed to be significantly inhibited [37].

*In vitro* studies of the ethanolic seed extract revealed its cytotoxic effect on a number of cancer cell lines such as breast cancer cell lines, prostate cancer cell lines, and pancreatic cancer cell lines [38]. Yet another study by Moalic et al. was done on diosgenin which showed its inhibitory effect on the human osteosarcoma cell line, that is, 1547 cell line [39]. The underlying mechanism was the arrest of cell cycle at G1 phase and the induction of apoptosis. Chemomodulatory effect of fenugreek seed extract was evaluated by Chatterjee et al. on two-stage mice skin carcinogenesis. DMBA and TPA were used to induce the skin tumor in mice which was inhibited by methanolic seed extract [40]. Chloroform seed extract also showed the effective killing of MCF-7 human immortalized breast cells through induction of apoptosis [41].

Devasena and Menon observed that fenugreek seeds in the diet inhibited colon carcinogenesis by modulating the activities of *β*-glucuronidase and mucinase [42]. The seed powder in the diet decreased the activity of *β*-glucuronidase significantly and prevented the free carcinogens from acting on colonocytes. Mucinase helped in hydrolyzing the protective mucin. This was attributed to the presence of fiber, flavonoids, and saponins.

### 2.4. Hypocholesterolemic Activity

Anticholesterol activity of fenugreek extracts has been well studied by the researchers all over the world. Studies have been performed* in vivo* and were not limited to the rats and mice as they were also performed on different species of rabbits. Singhal et al. studied that the inclusion of fenugreek seeds as a diet component for the mice aided in reducing cholesterol level up to 42% and 58% both in control group and in hypocholesterolemic group, respectively [43]. Another study was done to test the effects of fenugreek leaves on the cholesterol level. There was a reduction in total blood cholesterol, LDL, VLDL level, and triglycerides and there was an increase in HDL cholesterol level after the consumption of dried fenugreek leaves in Albino rabbits [44]. Presence of cholesterol in plasma is an indicator of coronary heart disease. Researchers have studied the effect of fenugreek seed extract on the lipid profile of plasma. Fenugreek seed administration and its extracts significantly decreased plasma cholesterol, triglyceride, and LDL cholesterol. However, HDL cholesterol level was found to be constant; that is, no effect was registered on it [45].

Isolation of a compound named GII from the seed extract of fenugreek with water was found to alter the level of serum lipids in diabetic induced rabbits. GII was able to reduce the total cholesterol level and increase HDL cholesterol which is an indicator of good cholesterol. There was a reduction in triacylglycerols, phospholipids, and free fatty acids [23]. The chemical constituents responsible for the activity are saponins, specifically diosgenin, galactomannan, and fiber.

### 2.5. Antigenotoxic Activity

Some researchers have used plant systems to examine the antigenotoxic effect of fenugreek. Chromosomal aberration assay in the* Allium* root is one of the most established assays to monitor the toxicity at the gene level. Root tip meristem cells of onion were treated with toxic chromium trioxide. Methanolic extract of the leaves of fenugreek showed dose-dependent decrease in chromosomal aberration in* Allium cepa* roots. Studies have also been done in microbial systems to observe the antimutagenic effect of fenugreek. Aqueous extract of fenugreek seeds inhibited the mutagenic activity of the direct acting mutagens against* Salmonella typhimurium* [46].

### 2.6. Anti-Inflammatory Activity

Fenugreek for past many years has been in use as a traditional medicine in several countries like Iran, southern India, and African countries as a remedy for inflammation and its related effects. The main chemical constituents responsible for the anti-inflammatory activity are alkaloids, saponins, and flavonoids. Sharififara et al. studied the* in vivo* effect of methanolic extract using cream based system. Inflammation in terms of edema was induced in Wistar rats using careegenan and anti-inflammatory effect was observed both by intraperitoneal administration and by the topical application in form of the cream [47].

Kawabata et al. studied the anti-inflammatory and antimelanogenic effect in* in vitro* system using human monocytic cell line (THP-1) [48]. Production of inflammatory cytokines such as IL-1, IL-6, and TNF-*α* was initiated using phorbol myristate acetate. Inhibitory action of fenugreek extract with methanol as a solvent system was observed with suppression in TNF-*α* production. The extract was further subjected to the isolation of bioactive compounds such as saponin and two other compounds which were also found to inhibit other cytokines like IL-1 and IL-6 along with TNF-*α*. The inhibitory effects were concentration dependent. Some contrasting results as compared to* in vitro* study were also evident in* in vivo* system as studied by Raju and others. TNF-*α* protein levels in the liver and plasma of obese rats were found to be upregulated when fenugreek was orally consumed. This is an indicator of opposing activity of fenugreek seed on the production of TNF-*α*. There are some complex mechanisms (e.g., digestion, uptake, and metabolism) by which orally administered substance(s) can modulate biological mediators* in vivo.* The opposing results in the production of TNF-*α* by fenugreek could be understood in terms of the difference between* in vitro* and* in vivo* systems [48]. Another study carried out by Sumanth et al. involved observing the anti-inflammatory effect against the ulcer production. Immersion stress and indomethacin were used to induce ulcer in rats. The aqueous extract of fenugreek seeds showed the antiulcer effect as calculated by the ulcer index [49]. The protective activity of the extract against ulcer could be attributed to its known antioxidant activity. Not only seeds but also antipyretic and anti-inflammatory activity of the leaves of* T. foenum-graecum* have been reported [50]. On similar lines, Ravichandiran and Jayakumari compared the anti-inflammatory activity of a bioactive compound isolated from fenugreek seed and leaves extracts and its aqueous extracts both in* in vivo* and in* in vitro* systems. It was observed that chloroform fraction of seeds and aqueous extract of leaves of fenugreek were effective against anti-inflammatory activity [51].

In a recent study, it was observed that when fenugreek was administered to diabetic mice, macrophage infiltration into adipose tissue was inhibited. Moreover, mRNA expression levels of inflammatory genes were also reduced. It is also suggested that fenugreek accelerates the wound healing process in rats injured in the posterior neck area due to its antioxidant potential [52].

### 2.7. Antimicrobial Activity

For past many years, scientists have been working on natural extracts to evaluate the antimicrobial properties for the development of novel therapeutics. Several plant systems such as* Coriandrum sativum, Curcuma longa*,* Citrus lemon*, and* Ocimum sanctum* have been studied by the scientists which exhibited antimicrobial action. Among various varieties of herbal extracts, fenugreek is also one of the candidates that have been tested for its activity against wide variety of microorganisms like bacteria, virus, and fungus [53–56].

Sensitivity of the extracts towards bacteria is dependent not only on the solvent system but also on the type of microorganisms as can be understood by the study done by Dash et al. [53]. A different strategy was employed to prepare the aqueous extract of fenugreek by Sheikhlar et al. Along with his coworkers, study was conducted on both the methanolic extract and aqueous extract against Gram-positive and Gram-negative bacteria. Contrary to the results obtained by other scientists, this study revealed performance by the methanolic extract, while the aqueous extract showed no activity at all concentrations. This study provided an insight into the dependence of antibacterial activity on the solvent system being chosen for the extract preparation [54]. Chandra et al. prepared the aqueous extract of fenugreek seeds and employed disc diffusion method to study the antibacterial effect on three bacteria, namely,* E. coli, P. putida, and M. furfur*. The extract was found to be effective against* E. coli* and* M. furfur* but showed no response against* P. putida* [55]. Crude extract of fenugreek seeds using methanol and acetone was tested against the four Gram-negative bacteria and it was observed that though the methanolic extract showed the broad range specificity towards various species,* S. typhi* showed resistance towards the acetonic extract. Also among all the four different strains* E. coli* was found to show the highest sensitivity towards acetonic extract, while methanolic extract showed an elevated response against* Pseudomonas* spp. It is also suggested that sprouted or the germinated seeds had enhanced antimicrobial activity specifically against* H. pylori* [56].

Secondary metabolites found in fenugreek seed extract possessed the antimicrobial activity as could be understood by various studies done by scientists. Similarly, these constituents can be found in the leaves of the fenugreek herb which can also exhibit the same property. Fungus being one of the microorganisms has also shown its sensitivity towards one of the proteins called defensin extracted from fenugreek leaves. Defensin not only inhibited the mycelial spread of* Rhizoctonia solani* but also inhibited spore germination and consequential hyphal growth of* Phaeoisariopsis *[57].

### 2.8. Gastroprotective Effect

In addition to various kinds of extracts, researchers have tried to extract oil from fenugreek seed which also possesses pharmacological properties. One such property is gastroprotective activity observed in oil extracted from fenugreek seed. The incidence of gastric ulceration, mean ulcer score, and ulcer index were found be significantly decreased in a group of mice subjected to indomethacin to induce ulcer. The decrease in the gastric ulcer can be attributed to phytic acid, saponins, and trigonelline found in the essential oil of fenugreek [58]. One of the studies reveals protective effect of aqueous extract of fenugreek seed against reflux esophagitis (RE) in rats and thus its potential to be used in clinical trial studies [59]. 

*Pharmacological Profile*. See Tables 1(a) and 1(b).

## 3. Discussion

With the onset of industrialization and progressive development, humans' reliability on machine has increased tremendously. But all this has come up with a very heavy cost of pollution and sedentary life style which resulted in rise in incidences of several diseases. Globally, cancer is one such disease that has become a big menace to humans. As per Indian population census data, the rate of mortality due to cancer in India was high and alarming with about 806,000 existing cases by the end of the last century [60]. After cardiovascular diseases, it is the most common cause of deaths among patients in India, responsible for mortality of about 300,000 deaths per year [61]. Lack of treatment and timely diagnosis along with poor availability of preventive methods and awareness are the major reasons for such high morbidity and mortality associated with cancer. Among the population in India, generally all types of cancers have been studied though the prevalence rate is highly variable. The types of cancer observed in Indian patients include the cancers of skin, lungs, breast, rectum, stomach, prostate, liver, cervix, esophagus, bladder, blood, and mouth. Both internal and external factors seem to be responsible for the cause of cancer. The internal factors are genetic mutations and hormonal, poor immune conditions and external or environmental factors include food habits, industrialization, over growth of population, pollution of air, water, and soil, excessive use of insecticides and pesticides, and many more. Since India is a growing economy, it cannot bear excessive burden of expensive treatment of such diseases and hence needs special attention on preventive natural and herbal treatment methods.

**Figure 1:**
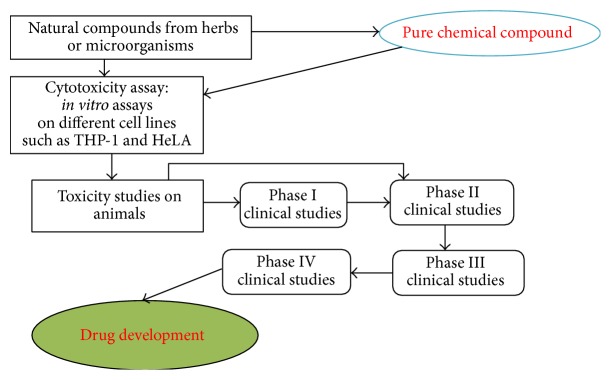
Herbal product as phytopharmaceutical: steps involved in the process of drug development.

Natural products derived from herbs and microorganisms have played a crucial role in numerous sectors for many centuries, one of them being treatment of diseases and health management. In spite of their widespread usage, some loopholes are there which need to be filled in. The crude extract of the herbs is a complex mixture of many compounds which makes it difficult to unravel the property of a specific compound. Therefore isolating a bioactive compound can act as a starting point for the drug development. Furthermore, emphasis should be given on the safety, efficacy, and toxicity of the preparation derived from medicinal plants. Amount of dose is also an important factor in toxicity and hence should be taken into consideration. As a result, the ultimate effect can be maximized and it will act as a boon for the health care industry.  Figure 1 can help us in understanding the process of drug development.

## 4. Conclusion

In this review, attempts have been made to describe the reported phytochemistry of fenugreek and its pharmacological uses. Because of its medicinal effects, many scientists consider fenugreek as a potential nutraceutical. The clinical uses of fenugreek can be attributed to the rich chemical constituents it possesses. These chemicals make it a strong candidate in every domain as they help in alleviating dependence on synthetic drugs as well as other expensive treatments to cure diseases. Further research and investigations can be done to isolate the bioactive compound from crude extract for drug development as it holds a promising future in the field of natural products to cure diseases. Proper research studies along with planned clinical trials are theneed of the hour so that the natural product from the plant can produce fruitful results for mankind.

## Figures and Tables

**(a):** 

Activity	Dose	Content	Mode of application	Vehicle	Parameters	Reference
Anticancer	200 mg/kg b.wt	Flavonoids	—	Aqueous and olive oil	Inhibition of the mammary hyperplasia	[35]
	100 mg/kg and 200 mg/kg b.wt	—	Intraperi- toneal	Ethanolic	Alterations of ascites cells	[36]
	25–800 mg/kg b.wt	Saponins, flavonoids, alkaloids, and galactomannans	Oral	Methanol	General: tumor incidence; biochemical: liver GSH and LPO	[39]
	2 g/kg b.wt	Saponins, flavonoids, and fiber	Oral	—	Inhibition of colon carcinogenesis by modulating glucuronidase and mucinase activity	[41]

Antidiabetic	0.44–1.74 g/kg·d	—	Oral	Aqueous	Reduction in blood glucose and improvement of hemorheological properties	[20]
	50 mg/kg b.wt and 100 mg/kg b.wt	—	Oral	Water	Decrease in blood glucose	[23]
	0.5 g/kg b.wt	—	Oral	Alcoholic	Reduction of blood glucose level	[62]
	1 g/kg b.wt	—	Oral	Aqueous and methanolic	Hypoglycemic effect	[63]

Anticholesterol/ hypocholesterolemic	0.44–1.74 g/kg·d	—	Oral	Aqueous	Reduction in blood lipid levels	[20]
	100 mg/kg b.wt	—	Oral	Water	Decrease in serum lipids	[23]
	0.5 g/kg	—	—	—	Decrease in triglycerides and total cholesterol	[43]
	30 or 50 g ethanol extract/kg b.wt	Saponins	Oral	Ethanol	Reductions in plasma cholesterol levels	[62]

Anti-inflammatory	50–200 mg/kg b.wt	—	—	Aqueous	Stimulatory effect on immune system	[64]
	150 mg/kg b.wt	—	—	Ethanolic	Effective against acute and chronic inflammation	[65]

Antioxidant	0.4 g/kg b.wt	—	Oral	—	Reduction in the level of serum MDA	[66]
	0.11 g/kg b.wt	—	—	—	Decrease in SOD activity	[67]
	0.4 g/kg b.wt	Flavonoids and polyphenols	Oral	Aqueous	Decrease in the level of MDA	[68]

Antimicrobial		Flavonoids		Aqueous	Inhibition of *E. coli* and *M. furfur*	[55]
				Aqueous, methanolic, and ethanolic	Methanolic and ethanolic extracts were effective against *E. coli*, *S. typhi*, and *S. aureus*	[69]

**(b):** 

Activity	Dose	Constituent	Vehicle	Parameters	Reference
Antioxidant	20–100 *μ*g/mL	Polyphenols	Methanol	Scavenging hydroxyl radical, DPPH, abts, changes in LPO, SOD, and inhibition of H_2_O_2_ induced lipid peroxidation	[27]
	25–200 mg/mL	Flavonoids and alkaloids	Ethanolic	Scavenging DPPH and inhibition of lipid peroxidation	[65]

Anticancer				Induction of apoptosis in HT-29 human colon cancer cells	[30]
		Diosgenin		Inhibition of cell proliferation in the human osteosarcoma 1547 cell line	[38]
		Diosgenin	Chloroform	Killed MCF-7 human immortalized breast cells	[40]
			Ethanolic	Cytotoxic to breast cancer cell line and prostate cancer cell line	

Antidiabetic	0.33 and/or 3.3 mg/mL	Saponin, sapogenin, diosgenin, and trigonelline	Ethanolic	Inhibition of glucose uptake	[70]
	1 gm/day	4-Hydroxyisoleucine	Hydro- alcoholic	Direct pancreatic *β*-cell stimulation, delayed gastric emptying, and inhibition of glucose transport	[71]
	5–20 mM	—	Aqueous	Reduction in *v*_max⁡_ of d(+)-glucose uptake	[72]

Anti-inflammatory		Saponin	Methanolic	Inhibition of TNF-*α* in THP-1 cell lines and restrained synthesis of melanin in murine melanoma B16F-1 cells	[48]
		Steroidal saponin	Aqueous	Peroxyl radical scavenging effects and reduction of release of ROS from inflamed mucosa	[73]

Antimicrobial	16–128 mg/mL	Essential oil	Methanolic and acetone	Antimicrobial activity against *Pseudomonas *spp. and *E. coli*	[53]
	100 mg	Defensin		Inhibits the mycelial spread of *Rhizoctonia solani*	[57]
